# The Relationships between Psychological Well-Being, Emotions and Coping in COVID-19 Environment: The Gender Aspect for Postgraduate Students

**DOI:** 10.3390/ijerph191610132

**Published:** 2022-08-16

**Authors:** Saule Sipaviciene

**Affiliations:** Department of Health Promotion and Rehabilitation, Lithuanian Sports University, 44221 Kaunas, Lithuania; saule.sipaviciene@lsu.lt

**Keywords:** postgraduate students, psychological well-being, emotions, coping, COVID-19

## Abstract

Background: Postgraduate students were exposed to the Coronavirus pandemic, and their study process changed from face-to-face to online. The purpose of this study was to analyze the impact of gender differences on emotions, coping strategies and psychological well-being (PWB) in the environment of the Coronavirus pandemic second wave (11 July 2020–30 June 2021). Methods: Ryff scale, MEQ Multidimensional emotion questionnaire, and brief COPE scale. The participants’ consisted of postgraduate students (74 female and 54 male). The study was conducted from 21 June 2022 to 28 June 2022. Results: Postgraduate students rated their PWB levels insignificantly in terms of gender. However, the individual components of this construct were evaluated as being significantly different in terms of gender. Females were more likely to feel negative emotions and had a harder time regulating these emotions than males. Female students were less likely than males to use problem-focused and avoidant-focused coping strategies. Conclusions: Postgraduate females were more affected than males by the Coronavirus pandemic. Females’ PWB was more concerned with emotions than males. Females were less likely than males to use problem-focused coping strategies.

## 1. Introduction

Psychological well-being can be defined as a multidimensional construct that includes six components: “self-acceptance, positive relation with others, autonomy, environmental mastery, purpose in life and personal growth” [1] (pp. 1070–1071).

The scholars defined the term coping as “the cognitions and behaviors, adopted by the individual following the recognition of a stressful encounter, that are in some way designed to deal with that encounter or its consequences” [2] (p. 7).

There is no common agreement on the definition of the term ˝emotions˝ [3]. However, many researchers agree that emotions involve a limited number of components and characteristics [4]. In this study, we will use the concept of the emotions construct and the totality of components, as presented by Klonsky et al. [5]. Thus, the construct of emotions includes ten discrete emotions, their frequency, intensity, and duration, emotion regulation, and the idea that emotions are subjectively and consciously perceived [5].

The COVID-19 pandemic changed many students’ living circumstances: contact with friends, overall substance use, and physical activity were decreased, but levels of depression, academic stress, and dissatisfaction with studies were increased, and symptoms of depression were significantly more pronounced in women [6]. Male students were less fortunate, with lower levels of psychological well-being compared to female students [7].

In the context of the Coronavirus pandemic, improved psychological well-being is influenced by a higher perception of meaningful living, and this differs in terms of gender [7]. Students with more work (business activity) and life experience after the COVID-19 pandemic reported lower levels of well-being and higher levels of depression and anxiety compared to pre-pandemic levels, but higher levels compared to all students [8].

The results of a previous study showed that females had poorer overall health perceptions, lower quality of life, more stress, and worse sleep quality than male students [9]. It has been revealed that coping strategies significantly predict both positive and negative emotions. Adaptive coping strategies help to maintain positive emotions and regulate denial of emotions [10,11].

Active coping strategies and family support appear among undergraduates as protective factors against a stressful environment, but passive coping strategies can exacerbate psychological problems for subjects [12]. 

During the COVID-19 pandemic, physical activity decreased, and daily habits and diet changed; this may have had a negative impact on psychological well-being, and female students rated these indicators significantly higher than male students [13].

The results of a previous study showed that two-thirds of students had sufficient knowledge of COVID-19 to be aware of the preventive measures associated with the pandemic but indicated that the COVID-19 outbreak affected their social, mental, and psychological well-being, and females were more affected than males [14].

The previous studies revealed positive associations between COVID-19-related difficulties and decreased levels of perceived coping with the pandemic. Coping strategies were also influenced by media coverage of the pandemic, and denial and substance use were significantly associated with poor communication, poor time planning, and disrupted studies during the Coronavirus pandemic [15,16].

In recent years, undergraduate students were more likely to use instrumental and emotional support and coping strategies than lower-year students. Their psychological well-being was at a higher level, and this was related to coping strategies such as positive reframing and humor [17].

In critical situations, a person can concentrate his or her mental and physical powers and balance challenging circumstances, and this balance is not easily disturbed by external events [18]. Achieving such a balance helps to combat the effects of a pandemic. Equilibrium is positively related to the components of the psychological well-being construct, such as autonomy, personal growth, purpose in life, self-acceptance, positive relations, and environmental mastery [19].

The previous study confirmed a negative association between COVID-19 fears and levels of psychological well-being. Students perceived that social support for well-being is very important in combating the effects of the Coronavirus pandemic [20].

It was found in previous research that, during the pandemic, students most commonly used the following coping strategies: acceptance, active coping, and physical activity. Female and graduate students used more coping strategies than male and bachelor’s students. Coping strategies were significantly correlated with psychological well-being [21].

An analysis of the studies mentioned above revealed that students were affected by stressors associated with the Coronavirus pandemic, had to switch to distance learning, had limited social contact and developed signs of depression, and that their psychological well-being deteriorated. Students chose different coping strategies. These negative symptoms were often more pronounced in female students than in male students [6,7,8,9,10,11,12,13,14,15,16,17,18,19,20,21].

However, the other researchers’ results do not sufficiently reveal the emotional expression of postgraduate students’, the peculiarities of their coping strategies, and their psychological well-being during the Coronavirus pandemic.

The purpose of the present study was to analyze the impact that gender differences have on emotions, coping strategies, and the psychological well-being of postgraduate students in the Coronavirus pandemic environment.

**Hypothesis** **1** **(H1).**  *Postgraduate female students are more affected by the* *stressors associated with the Coronavirus pandemic, but their level of psychological well-being is higher than that of male students.*

## 2. Materials and Methods

### 2.1. Participants and Procedures

The participants for this study were selected using a purposive sampling method from postgraduate students of social science study programs. The sample consisted of 74 female and 54 male (a total of 128) postgraduate students after the end of lockdown (after 30 June 2021) from three state universities operating in the city of Kaunas, Republic of Lithuania. All students participated in the study voluntarily, with no financial incentive, and they were informed of their right to terminate their participation in this study at any time. The research was conducted following the principles of reliability, honesty, respect, and accountability. The Ethics Committee of Social Sciences Research of the Lithuanian Sports University issued a permit to conduct this research, and it meets the ethical and legal requirements in Lithuania, where the research was conducted. Study participants were informed about the purpose of the study, the study organization, and the study process. Participants were also informed that their personal data would not be collected, and data collected through research instruments would be processed and stored following the requirements of the Personal Data Protection Code. Participants completed a questionnaire, which initially required a response to the statements “I agree to participate” or “I disagree to participate”, and there was no time limit. When asked about completing the questionnaire, the researcher provided personal counseling, but not when choosing answers to items in the questionnaire. The questionnaire survey was carried out during classes after prior coordination of the research time with students and teachers. Before this study, there were two waves of COVID-19 in Lithuania. The first lockdown was from 16 March 2020 to 17 June 2020, and the second lockdown was from 7 November 2020 to 30 June 2021. These dates were set by the government. This survey was conducted from 21 June 2022 to 28 June 2022.

### 2.2. Methods

The study questionnaire consisted of a sociodemographic part (gender, age, year of study), a Ryff Scale for measuring psychological well-being [1], the multidimensional emotion questionnaire [5], and a brief COPE scale to evaluate coping strategies [22,23].

#### 2.2.1. Psychological Well-Being Scale

The scale for measuring psychological well-being consists of 54 items, divided into nine items in each of the six subscales: autonomy, environmental mastery, personal growth, positive relationships with others, purpose in life, and self-acceptance.

Subjects were asked to rate the items on the Likert scale from 1 to 6:1 = strongly disagree, 2 = disagree somewhat, 3 = disagree slightly, 4 = agree slightly, 5 = agree somewhat, and 6 = strongly agree. The higher the total score, the higher the subject’s psychological well-being. The overall psychological well-being score was calculated by obtaining the average of the outcomes in the items evaluated by the participants [1].

Cronbach alpha in this study of subscales was autonomy 0.70, environmental mastery 0.63, personal growth 0.63, purpose in life 0.64, and self-acceptance 0.71.

#### 2.2.2. Multidimensional Emotion Assessment Questionnaire

The multidimensional emotion questionnaire includes five positive emotions (happy, excited, enthusiastic, proud, and inspired) and five negative emotions (sad, afraid, angry, ashamed, and anxious). The frequency, intensity, and persistence of emotions, as well as the difficulty of regulating emotions, were assessed for each of these discrete emotions on a 5-point scale: 1. How often? About once per month or less; About once per week; About once each day; 2 or 3 times each day; More than 3 times each day. 2. How intense? Very low; Low; Moderate; High; Very high. 3. How long-lasting? Less than 1 min; 1–10 min; 11–60 min; 1–4 h; Longer than 4 h. 4. How easy to regulate? Very easy; Easy; Moderate; Difficult; Very difficult. 

The scores for indicators such as positive frequency, positive intensity, positive persistence, and positive overall were calculated by summing the respective estimates. The scores for indicators such as negative frequency, negative intensity, negative persistence, and negative overall were analogously calculated. Estimates of positive overall emotional regulation and negative overall emotional regulation were calculated by summing the scores for the difficulty of discrete emotional regulation. The Cronbach alpha of original scales ranged from 0.61 to 0.85 [5].

Cronbach alpha in this study for discrete emotions subscales was happy 0.70, sad 0.64, afraid 0.63, excited 0.66, angry 0.72, ashamed 0.62, enthusiastic 0.64, proud 0.71, anxious 0.66, and inspired 0.74.

#### 2.2.3. Coping Strategies Measure Scale

The brief COPE scale consists of 28 items and 14 subscales, with two items per subscale [22]. Scales singled out and verified by the developer (Cronbach’s alpha coefficient of the original scales ranged from 0.50 to 0.82 [22]. The participants were asked to rate the items on the scale in a four-point system 1—I haven’t been doing this at all; 2—A little bit; 3—A medium amount; and 4—I’ve been doing this a lot. The sub-scale scores were calculated as the averages of the estimates of the two appropriate items provided by the participants.

Scales can be divided into three higher-level super-scales: problem-focused coping, emotion-focused coping, and avoidance coping [24]. Scores for problem-focused coping were calculated as the average of the ratings of the eight corresponding items. Scores for emotion-focused coping were calculated as the average of the ratings for the 12 items in question. Scores for avoidant-focused coping were calculated as the average of the ratings of the eight corresponding items.

Cronbach’s alpha coefficients in this study for subscales were as follows: active coping 0.61; using instrumental support 0.68; positive reframing 0.61; planning 0.67; using emotional support 0.69; venting 0.67; humor 0.72; acceptance 0.62; religion 0.74; self-blame 0.63; self-distraction 0.68; denial 0.63; substance use 0.75; and behavioral disengagement 0.64.

### 2.3. Data Analysis

Research data were analyzed using IBM SPSS for Windows 22.0. The values of skewness and kurtosis of all variables of scales or subscales ranged from 1.67 to −1.558 (the limiting values ranged from 2 to −2), so the distribution of all variables does not significantly differ from the normal distribution and the Student’s *t* criterion can be used for comparisons between the means of scores [25]. Pearson correlation coefficients between the components of the psychological wellbeing, emotions, and coping strategies constructs were calculated. The statistical significance level was set at *p* < 0.05.

## 3. Results

The scores for the psychological well-being construct components are presented in Table 1. The psychological well-being construct of personal growth was rated the highest (3.71) and the environment mastery received the lowest score (3.37) from female students. Male students gave the highest score for the component of positive relations (3.77) and purpose in life was given the lowest score (2.98). There were statistically significant differences in the evaluations of all components by females and males (*p* < 0.05). Females rated the components of personal growth, autonomy, and purpose in life higher than males, and males rated positive relations, self-acceptance, and environmental mastery higher than females. The overall assessment of psychological well-being did not significantly differ between females and males.

Female and male students rated coping strategies differently (Table 2). Male students rated active coping, use of instrumental support, positive reframing, and planning statistically significantly higher (*p* < 0.05) than female students. The evaluations of the other coping strategies did not significantly differ according to gender.

Male students rated problem-focused coping and avoidant-focused coping statistically significantly higher than female students. Female students rated emotion-focused coping strategy higher than male students, but the difference in scores was not significant. Male students rated avoiding coping significantly higher than female students. Higher scores indicated higher levels of these coping strategies. 

Male students rated their positive emotions statistically significantly (*p* < 0.05) higher than female students, although the ratings for emotions such as happy and enthusiastic did not significantly differ (Table 3). Female students rated negative emotions such as afraid, angry, and anxious statistically significantly higher (*p* < 0.05) than male students, while male students only rated the emotion sad higher than female students. The scores for negative emotions and ashamed emotions did not significantly differ in terms of gender. Male students rated the components of positive frequency, positive intensity, and negative persistence statistically significantly higher (*p* < 0.05) than female students. The estimates of components such as positive persistence, negative frequency, and negative intensity did not significantly differ. Both positive and negative emotions were more difficult for female students to regulate. Female student scores for both cases of emotion regulation were statistically significantly higher (*p* < 0.05) than male student. The positive overall ratings for emotional components were higher for males and negative overall ratings were higher for female students than for male students, but not significantly.

Estimates given by female students regarding difficulties regulating positive emotions (Table 4) decreased in the following order: excited, enthusiastic, inspired, happy, and proud. The regulation of positive emotions such as happy, enthusiastic, and proud was more difficult for female than male students, and the difference in means was statistically significant (*p* < 0.05). The estimates regarding difficulties in the regulation of the positive emotions excited and inspired did not significantly differ in terms of gender. Overall regulation of positive emotions was more difficult for female than male students, and the difference was statistically significant (*p* < 0.05).

Negative emotion scores given by female students can be arranged in descending order according to the difficulty in their regulation: sad, afraid, anxious, angry, and ashamed. It was more difficult for female students to regulate negative emotions such as afraid, anxious, angry, and ashamed, than male students, and the difference in the corresponding emotion scores was statistically significant (*p* < 0.05). Overall regulation of negative emotions was more difficult for female than male students, and the difference was statistically significant (*p* < 0.05).

The overall negative emotion regulation score for female students (15.00 ± 1.89) was significantly higher than overall positive emotion regulation scores (12.92 ± 1.87), (*t* (146) = −6.737, *p* = 0.000), which suggests that female students rated negative emotions as being more difficult to regulate than positive emotions. The overall negative emotion regulation score for male students (12.17 ± 1.71) was not significantly higher than overall positive emotion regulation scores (11.39 ± 2.83), (*t* (106) = −1.727, *p* = 0.087). 

The components of the psychological well-being construct and the overall psychological well-being Pearson correlation coefficients were calculated, along with the components of the emotion construct and the coping strategy construct. Statistically significant Pearson correlation coefficients were found between the psychological well-being construct and its components and emotion and coping constructs components for female and male students, which are separately presented in Table 5.

The psychological well-being construct component autonomy showed a statistically significant relationship with the coping strategy of self-blame (r = −0.326) in female students, but there was no significant coefficient for male students. The component of environment mastery showed a significant correlation coefficient with the emotion happy (r = −0.238) for female students and with the coping strategies of planning (r = 0.268), and venting (r = 0.379) for male students. Personal growth was only significantly related with positive reframing (r = −0.274) for male students. The component of positive relations was statistically significantly related to the regulation of anxiety (r = 0.297) and negative emotions (r = 0.287), positive reframing (r = 0.279), and emotion-focused coping for female students, and there was no significant coefficient for male students. The component of purpose in life was significantly related to the emotions sad (r = 0.230) and anxious (r = 0.245) for female students. The psychological well-being component of self-acceptance was significantly related to the emotion inspired (r = 0.284), indicative of the regulation of anger (r = −0.229), and was significantly related to the negative emotion regulation (r = −0.248) for female students, and significantly related to the emotion inspired (r = 0.382) for male students. Overall psychological well-being was significantly positively related to the emotion inspired (r = 0.239) and regulation of enthusiasm (r = 0.244) for female students, and negatively related to the emotion happy (r = −0.279), and positively related to the coping strategy of positive reframing (r = 0.311) for male students. 

## 4. Discussion

This study investigated the peculiarities of the relationships between psychological well-being, emotions and coping in postgraduate students in the COVID-19 environment in term of gender. 

The results of this study show that psychological well-being is higher in female students than male students, but the difference is insignificant (*p* > 0.05). The data obtained by various researchers are quite contradictory regarding the psychological well-being of females. This may be related to their multifaceted activities, as other researchers note that intensive activity can have a positive impact on psychological well-being [26].

A good level of communication is thought to help maintain a higher level of psychological well-being [27]. An effective coping strategy is vital to maintaining high levels of psychological well-being under extreme conditions [28]. However, the COVID-19 environment usually limited face-to-face communication between students.

Other researchers found that women’s mental health rates were lower than those of the general population due to exposure to Coronavirus during the pandemic and many factors. The importance of social connections in combating the environmental impact of COVID-19 has been clarified and is particularly relevant for women [29,30].

The results of this study revealed that female students value their autonomy, personal growth, and purpose in life higher than male students. According to other researchers, the Coronavirus pandemic affected female students more, with an increased level of anxiety being one of the discrete emotions noticed among students during the Coronavirus pandemic, especially among female students, compared to the pre-pandemic period [31,32]. Students who felt high levels of psychological well-being were more likely to choose active coping styles, while those with lower PWB were more likely to choose avoidance-type coping strategies [33].

It has been observed that the longer the pandemic period lasted, the more frequent the depressive syndromes became, especially in the female groups [34]. Pandemic environmental factors are expected to affect student well-being and may have uncertain effects in the future [35]. Students’ psychological well-being was also influenced by their social assistance, as shown in the previous study [36].

Satisfaction with well-being depends on many factors; it was found that the levels of satisfaction during the pandemic were very different when comparing the results of a survey of students in nine countries [37]. Postgraduate students indicated being satisfied with their living standards more often than undergraduates, and the weaker the effect of the Coronavirus pandemic, the more patented the students’ well-being [37]. These differences can be explained by individual genetic differences that determine a more or less positive human condition, in addition to influential cultural norms [38].

It has been revealed that the psychological well-being of undergraduate students can affect their further professional success and future position in society, so it is very important to take care of students during pandemics [39].

Decreases in the level of psychological well-being in university and college students due to exposure to COVID-19 have been reported by other researchers [40,41].

Perceived life changes associated with the limited social contact due to COVID-19 resulted in increased psychosocial distress, anxiety, depressive symptoms, and lower levels of psychological well-being [42,43].

Competencies are very important for students’ perceptive psychological well-being, but during the pandemic lockdown, there has been a transformation of studies from face-to-face to online. This required students to master new technologies for their studies, which contributed to a decline in their level of psychological well-being [28,44]. 

In this study, female students rated three of the six components of the psychological well-being construct higher than males, and male students rated the other three components of the construct higher (*p* < 0.05) than female students. However, the overall level was higher for female students, but not significantly higher than that of men. Other researchers also point out that summarizing the results of studies in 166 countries found that females only valued psychological well-being slightly higher than males [45]. 

The previous study showed that properly chosen coping strategies and positive emotions positively influence psychological well-being [46]. The results of this study confirmed the results of the above-mentioned study, showing that psychological well-being is related to emotions and coping strategies, as correlations of varying strength were revealed between the components of the constructs of psychological well-being, emotions, and coping strategies.

This study discloses the coping strategies used by female students and male students, providing an important complement to the pandemic knowledge, as other researchers point to the fight against stress and depressive symptoms and add to the lack of knowledge of coping strategies that are perceived to be effective in global pandemics [47]. The results of this study showed that female students rated emotion-focused coping strategies higher than male students and it was more difficult for female students to regulate both positive and negative emotions. Male students rated problem-focused coping strategies higher than female students, and it was easier for them to regulate their emotions. The results of this study revealed that female students rated the coping strategies of venting, using informational support, acceptance, and humor in descending order, while the lowest scores were given to the coping strategies of denial, substance use, self-distraction, positive reframing, and self-blame. Male students evaluated coping strategies in a different order. The top scores were active coping, use of informational support, planning, positive reframing, and venting. Thus, only the informational support coping strategy was among the top five strategies used by both female and male students. 

The previous study showed that the most common and least frequently used coping strategies, without disaggregating them by gender, were self-acceptance, planning, and emotional support strategies for the most common, and substance use, denial, behavioral disengagement coping strategies for the least common [48]. Among the problem-focused coping strategies, females and males more often used the active coping strategy, and among emotion-focused coping strategies, they most often used the venting strategy. Those using the active coping strategy were found to have higher levels of psychological well-being [49]. 

In this study, females and males gave the denial coping strategy the lowest score, and thus found it the least appropriate, with scores of 3.46 and 3.65, respectively. Those using the denial coping strategy may experience signs of depression [50]. 

In this study, male students rated highest in problem-focused coping and females were rated highest in emotion-focused coping strategies. The avoidant coping strategy received the lowest estimates from both groups. It has been observed that the choice of coping strategy is related to assessment of the situation. Previous studies showed that problem-focused strategies are more commonly used in situations where factors can be controlled, and emotion-focused strategies are used when factors are difficult to manage [51]. 

This study found that postgraduate female and male students found it more difficult to regulate negative emotions than positive ones. Other authors suggest that the predominance of negative emotions may have been related to the restrictions imposed during the Coronavirus pandemic, including online studies, and difficulties regulating emotions can negatively affect psychological well-being [51,52,53].

Consistent with the results of this study, despite the Coronavirus pandemic environment, postgraduate students felt positive emotions more often and more intensely than negative emotions. However, negative emotions lasted longer than positive ones, especially for males. All emotions and coping constructs were associated with a stronger or weaker relationships with psychological well-being, but the correlation coefficients were of different strengths for females and males. Statistically significant relationships were found with the emotion inspired and the regulation of enthusiasm for female students, and for male students, there was a statistically significant relationship between the emotion happy and the coping strategy of positive reframing.

The results of this study partially confirmed the hypothesis. Female students rated their psychological well-being as being higher than that of male students, but not significantly. Female students used the emotion-focused coping strategy more often than males. Negative emotions were rated higher by female than male students, and female students also found it more difficult to regulate negative emotions.

### 4.1. Limitations of This Study

One of the limitations of this study was that it was a single-section study, and it was not possible to compare the data from this study with data from the pre-pandemic period. Although the lockdown has already been reversed, due to some remaining limitations, the number of subjects is limited, so the results of the study cannot be extrapolated, not only for postgraduate, but for all students nationwide. Another limitation of the survey results is the peculiarities of the data collection instruments, as the respondents themselves completed the questionnaire and scales. The study needed to be completed as soon as possible after the lockdown, as most people tend to forget about negative influences quickly and have a more positive view of the past. A third limitation of the study is the small sample size.

The strength of this study, from the researchers’ perspective, lies in the fact that all components of the constructs of psychological well-being, emotions, and coping strategies were evaluated separately from a gender perspective.

### 4.2. Future Research

Future studies could examine how people’s perceptions of the effects of the Coronavirus pandemic change over time, especially as new waves of COVID-19 strains are expected. Students will have gained experience in online study and living in a restricted social network. It would also be appropriate to extend the study to university and college students.

## 5. Conclusions

The effects of the Coronavirus pandemic on female and male postgraduate students differed according to gender. Assessments of the overall level of psychological well-being differed insignificantly between female and male students, but assessments of the components of this construct significantly differed in terms of gender. Three components (autonomy, personal growth, and purpose in life) were rated higher by female students, while the other three (environmental mastery, positive relation, and self-acceptance) were rated higher by male students. During the pandemic, male students were significantly more likely to use (overestimate) all problem-focused coping strategies (active coping, use informational support, positive reframing, and planning) than female students. However, female students were significantly less likely to use (underestimate) avoidant-focused coping strategies. Evaluations of emotion-focused coping strategies differed insignificantly in terms of gender. 

Female students found it much harder to regulate negative emotions than male students. Female students’ psychological well-being is more related to emotions than male students. Male students’ psychological well-being is significantly associated with the coping strategy of positive reframing.

## Figures and Tables

**Table 1:** The scores of psychological well-being construct components in terms of gender *.

Component of Construct	Female (*n* = 74)		Male (*n* = 54)		Student *t*	*p*
	*M*	*SD*	*M*	*SD*		
Autonomy	3.56	0.547	3.34	0.604	2.097	0.038
Environmental mastery	3.37	0.420	3.63	0.526	−3.157	0.002
Personal growth	3.71	0.524	3.43	0.525	2.933	0.004
Positive relations	3.58	0.465	3.77	0.443	−2.329	0.021
Purpose in life	3.45	0.465	2.98	0.538	4.357	0.000
Self-acceptance	3.43	0.573	3.69	0.551	−2.559	s
Psychological well-being	3.51	2.13	3.49	0.223	0.634	0.524

* Note: Ryff scale (Ryff, 1989) [1].

**Table 2:** Scores of coping construct components in terms of gender *.

Component of Construct	Female (*n* = 74)		Male (*n* = 54)		Student *t*	*p*
	*M*	*SD*	*M*	*SD*		
Active coping	5.51	1.14	6.43	1.08	−4.86	0.000
Use informational support	5.18	1.40	6.30	1.14	−4.827	0.000
Positive reframing	4.43	1.62	5.37	1.56	−3.285	0.001
Planning	4.99	1.41	5.89	1.21	−3.796	0.000
Use emotional support	4.91	1.66	5.17	1.60	−0.894	0.373
Venting	5.58	1.41	5.24	1.34	1.372	0.172
Humor	5.04	1.54	5.07	1.44	−0.125	0.901
Acceptance	5.12	1.73	4.72	1.58	1.338	0.183
Religion	4.96	1.50	4.82	1.64	0.518	0.186
Self-blame	4.93	1.68	4.94	1.89	−0.038	0.970
Self-distraction	3.88	1.07	4.20	1.11	−1.673	0.097
Denial	3.46	0.95	3.65	1.03	−1.063	0.287
Substance use	3.81	0.90	4.03	1.03	−1.321	0.189
Behavioral disengagement	4.37	1.40	4.65	1.40	−1.129	0.261
Problem-focused coping	5.03	0.464	6.00	.492	−11.38	0.000
Emotion-focused coping	5.09	0.586	4.99	0.721	0.830	0.408
Avoidant-focused coping	3.88	0.542	4.13	0.631	−2.462	0.015

* Note: MEQ Multidimensional emotion questionnaire (Klonsky et al., 2019) [5].

**Table 3:** Scores of the emotion variables in terms of gender *.

Component of Emotion Construct	Female (*n* = 74)		Male (*n* = 54)		Student *t*	*p*
	*M*	*SD*	*M*	*SD*		
Positive emotions						
Happy	9.23	1.83	9.39	2.09	−0.460	0.647
Excited	8.59	2.26	9.35	1.63	−2.090	0.039
Enthusiastic	8.41	2.16	8.42	2.54	−0.049	0.961
Proud	8.23	2.16	9.37	1.78	−3.175	0.002
Inspired	8.05	2.49	9.35	2.26	−3.029	0.003
Positive frequency	15.45	2.79	17.09	2.78	−3.308	0.001
Positive intensity	14.12	2.93	15.37	2.77	−2.435	0.016
Positive persistence	12.95	2.81	13.43	2.84	−0.951	0.344
Positive Overall	42.51	5.15	45.89	5.41	−3.484	0.000
Negative emotions						
Sad	7.88	2.22	10.43	1.31	−7.523	0.000
Afraid	8.69	1.70	7.91	2.01	2.380	0.019
Angry	9.18	2.04	8.02	1.97	3.215	0.002
Ashamed	8.69	2.40	8.43	2.04	0.651	0.516
Anxious	8.66	2.34	7.61	2.28	2.540	0.012
Negative frequency	14.51	2.76	13.65	2.84	1.729	0.086
Negative intensity	13.89	3.33	12.85	2.67	1.893	0.061
Negative persistence	14.69	3.05	15.89	3.20	−2.151	0.033
Negative Overall	43.10	5.25	42.39	4.27	0.812	0.412

* Note: Brief COPE scale (Carver, 1997) [23].

**Table 4:** Data on emotions regulation difficulties in terms of gender *.

Component of Emotion Construct	Female (*n* = 74)		Male (*n* = 54)		Student *t*	*p*
	*M*	*SD*	*M*	*SD*		
Positive emotions						
Happy	2.284	0.786	1.944	0.834	2.352	0.020
Excited	3.284	1.092	3.093	1.508	0.832	0.407
Enthusiastic	2.578	0.704	2.019	0.901	3.871	0.000
Proud	2.230	0.713	1.778	0.817	3.331	0.001
Inspired	2.554	0.967	2.556	1.127	−0.008	0.994
Overall positive emotions regulation	12.92	1.87	11.39	2.83	3.679	0.000
Negative emotions						
Sad	3.419	0.965	3.148	1.323	1.339	0.183
Afraid	3.108	0.959	2.482	1.059	3.496	0.001
Angry	2.770	0.673	2.259	0.873	3.739	0.000
Ashamed	2.662	0.849	2.222	0.883	2.848	0.005
Anxious	3.041	0.784	2.056	0.738	7.195	0.000
Overall negative emotions regulation	15.00	1.89	12.17	1.71	2.655	0.009

* Note: Brief COPE scale (Carver, 1997) [23].

**Table 5:** The correlation coefficient between psychological well-being and components of emotion construct.

Component of Construct		Autonomy	Environment Mastery	Personal Growth	Positive Relations	Purpose in Life	Self- Acceptance	PWB
Female								
Emotions	Happy	−0.063	−0.238 *	0.038	0.134	0.024	0.022	0.025
	Sad	−0.107	−0.057	0.112	0.162	0.230 *	−0.110	0.132
	Anxious	−0.076	−0.099	0.053	0.087	0.245 *	0.005	0.126
	Inspired	0.150	0.151	0.155	−0.054	−0,115	0.284 *	0.239 *
	Anxious regulation	0.063	−0.015	−0.015	0.297 **	0.014	−0.083	0.097
	Angry regulation	0.144	0.123	0.165	0.169	0.123	−0.229 *	0.181
	Enthusiastic regulation	−0.009	0.081	0.226	−0.032	0.175	0.035	0.244 *
	Negative emotion regulation	0.060	0.059	−0.029	0.287 *	−0.076	−0.248 *	−0.001
Coping strategy	Self-blame	−0.326 **	−0.122	0.055	0.021	0.131	−0.098	−0.113
	Positive reframing	−0.115	−0.043	0.107	0.279 *	−0.076	−0.141	−0.196
	Emotion focused coping	−0.073	−0.226	−0.059	0.275 *	0.023	0.111	−0.002
Male								
Emotions	Happy	−0.172	0.014	−0.162	0.060	−0.185	−0.053	−0.279 *
	Inspired	0.048	0.065	−0.104	−0.044	−0.092	0.382 **	−0.190
Coping strategy	Positive reframing	−0.014	−0.232	−0.274 *	−0.141	0.026	0.021	0.311 *
	Planning	−0.196	0.268 *	0.077	0.071	0.218	−0.062	0.222
	Venting	0.138	0.379 **	0.016	−0.012	−0.304 *	0.151	0.105
	Emotion support	0.021	0.048	0.030	0.311 *	−0.020	0.054	−0.061
	Self-distraction	−0.055	0.213	0.081	−0.277 *	0.147	0.134	−0.114

Notes: * *p* < 0.05 (two-tailed); ** *p* < 0.01 (two-tailed).

## Data Availability

The datasets collected and analyzed during the current study are available from the corresponding author on reasonable request. All survey data are password protected.
